# Metabolic analysis of acute appendicitis by using system biology approach 

**Published:** 2018

**Authors:** Homayoun Zojaji, Majid Rezaei Tavirani, Vahid Mansouri, Ali Seyed Salehi, Reza Mahmoud Robati, Elena Lak

**Affiliations:** 1 *Gastroenterology and Liver Diseases Research Center, Research Institute for Gastroenterology and Liver Diseases, Shahid Beheshti University of Medical Sciences, Tehran, Iran*; 2 *Department of Surgery, Faculty of Medicine, Iran University of Medical Sciences, Tehran, Iran*; 3 *Proteomics Research Center, Faculty of Paramedical Sciences, Shahid Beheshti University of Medical Sciences, Tehran, Iran*; 4 *Foodborne and Waterborne Diseases Research Center, Research Institute for Gastroenterology and Liver Diseases, Shahid Beheshti University of Medical Sciences, Tehran, Iran*; 5 *Skin Research Center, Shahid Beheshti University of Medical Sciences, Tehran, Iran *; 6 *Imam Hossein Hospital, Shahid Beheshti University of Medical Sciences, Tehran, Iran*

**Keywords:** Appendicitis, Gene, Biomarker

## Abstract

**Aim:**

Introducing possible suitable compound as diagnostic agent in appendicitis is aim of this investigation.

**Background:**

Appendicitis diagnosis is a difficult step in treatment of disease due to complex abdominal pain signal which may be refer to the non-appendicitis pain.

**Methods:**

Gene expression profiles of children with non-preforated appendicitis in comparison with the samples with non- appendicitis abdominal pain are analysis via protein – protein interaction (PPI) and the critical compounds are introduced by STITCH.

**Results:**

Ten compounds including including MgATP, glycerol, MgADP, calcium ions, chloride, magnesium, phosphate, sulphate, acetate, and sodium are introduced as possible biomarker panel to differentiate appendicitis from the other abdominal pains.

**Conclusion:**

A laboratory method such as flame photometry based on metal detection for diagnosis of appendicitis is possible, however more investigations are required.

## Introduction

 Appendicitis as one of the poorly recognized abdominal pain is common in all ages worldly (1). The annual incident of this emergency condition is 1.1 cases per 1000 individuals (2) with highest rate of abdominal surgery in children (3). The diagnosis, screening and treatment for this condition is remained complicated (1) due to heterogeneity of clinical manifestation and lack of potential biomarkers (4). What is more, one of the drawbacks of current clinical diagnostic methods is the usage of tomography that imposes notable radiation contact (5). Additionally, there are some reports regarding misdiagnosis of appendicitis that resulted in removing healthy appendix with rate of 17% to 28% outside the U.S. and Western Europe (5). Thus, the requirement for detecting putative biomarkers in this regard concluded in different molecular investigations (6). These studies could introduce elements that possibly upgrading clinical evaluations (4, 7). In view of this fact, there are some high throughput studies including genomics, proteomics, and metabolomics (4, 8, 9). In addition, to gain more reliable and accurate candidates, one way is to study these suggested biomarkers by system biology in terms of interaction values. Protein-protein interaction (PPI) network analysis could be beneficial in adding more knowledge about the identified biomarkers and their corresponding biological processes and filtering the most promising ones for advancing clinical assessments (10, 11). In a way that, the PPI are key assets of accelerating a biological response to a certain condition of a cell. Modifications in these interactions could change the vital characteristic of that cell. In another world, abnormal conditions such as a disease state could occur. The participations of these communicating proteins are critical in the matter of dysregulations. In fact, any significant expression alteration in these agents may be responsible for abnormal interactome and ultimately for the disease condition (12). Consequently, for the analysis and better understanding of the mechanisms underlying Appendicitis, PPI network study could be favorable via transcriptome profile examinations. 

## Methods

Gene expression profiles of 4 acute appendicitis and 17 inguinal hernia (as controls); GSE83091/GPL10558, were retrieved from GEO. The samples were matched via box plot analysis and the comparable ones were selected four further analysis. Numbers of 250 top significant DEGs were considered and the significant and characterized ones were selected to analyze via PPI network analysis. After adding 100 relevant proteins or compounds, the queries were included in a interacted unit by STITCH a plugin of Cytoscape software (13). The top 10% of nodes based on degree value were determined as hubs. All interacted compounds were selected as relevant compounds which are involved in appendicitis. The top compounds which were common with top hubs were identified as critical compounds related to appendicitis. P-value less than 0.05 and fold change more than 2 and less than 0.5 were regarded. 

**Table 1:** Top 10% nodes of appendicitis network based on degree value are presented as hubs

R	Name	description	node type	Degree	BC	CC
1	MgATP		compound	111	0.044593	0.690141
2	glycerol		compound	103	0.064153	0.671233
3	MgADP		compound	103	0.033205	0.668942
4	calcium ions		compound	97	0.039272	0.646865
5	chloride		compound	91	0.040757	0.636364
6	magnesium		compound	91	0.03123	0.636364
7	phosphate		compound	86	0.014685	0.636364
8	sulphate		compound	84	0.030589	0.622222
9	acetate		compound	83	0.02295	0.622222
10	MAPK3	mitogen-activated protein kinase 3	protein	81	0.016042	0.624204
11	PIK3CA	phosphatidylinositol-4,5-bisphosphate 3-kinase, catalytic subunit alpha	protein	79	0.009832	0.604938
12	AKT1	v-akt murine thymoma viral oncogene homolog 1	protein	79	0.015188	0.6125
13	MAPK1	mitogen-activated protein kinase 1	protein	78	0.013199	0.618297
14	carboxy		compound	77	0.011449	0.61442
15	sodium		compound	77	0.031398	0.608696
16	distilled water		compound	73	0.012154	0.603077
17	GAPDH	glyceraldehyde-3-phosphate dehydrogenase	protein	72	0.008353	0.590361
18	Zn(II		compound	72	0.021814	0.593939
19	HRAS	v-Ha-ras Harvey rat sarcoma viral oncogene homolog	protein	71	0.021469	0.597561
20	guanosine trip.		compound	70	0.007893	0.590361

## Results

**Figure 1:**
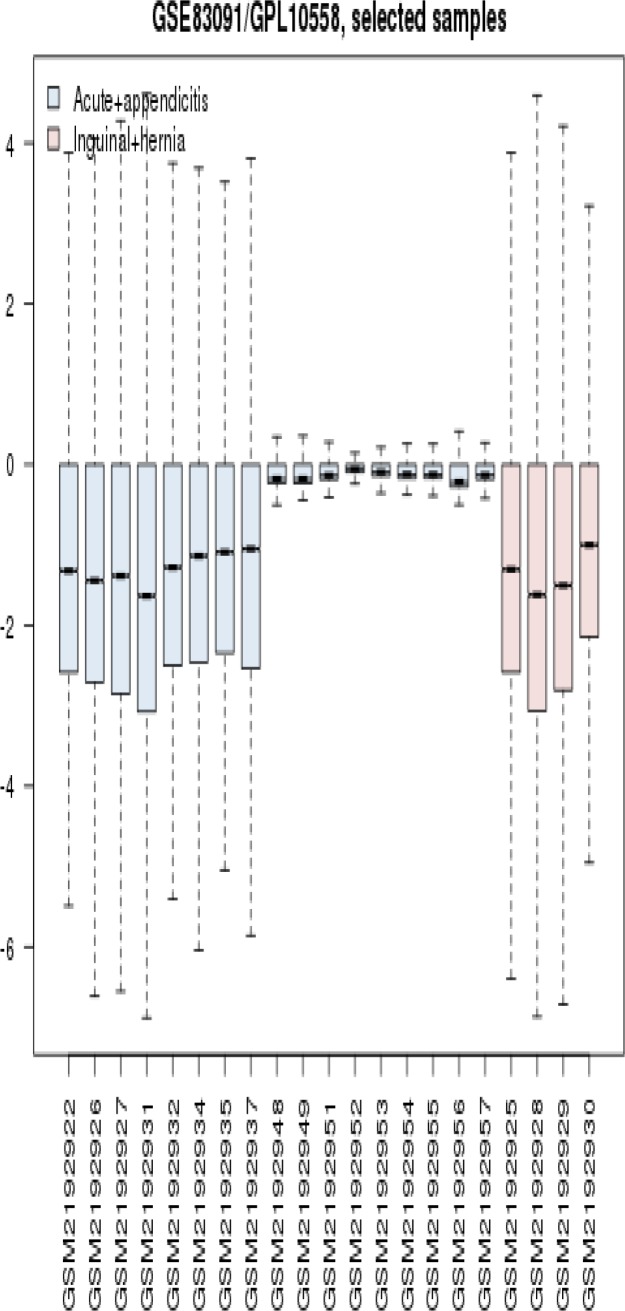
Box plot illustration of gene profiles of 4 acute appendicitis and 17 inguinal hernia samples. The analyzed profiles are not comparable

**Figure 2:**
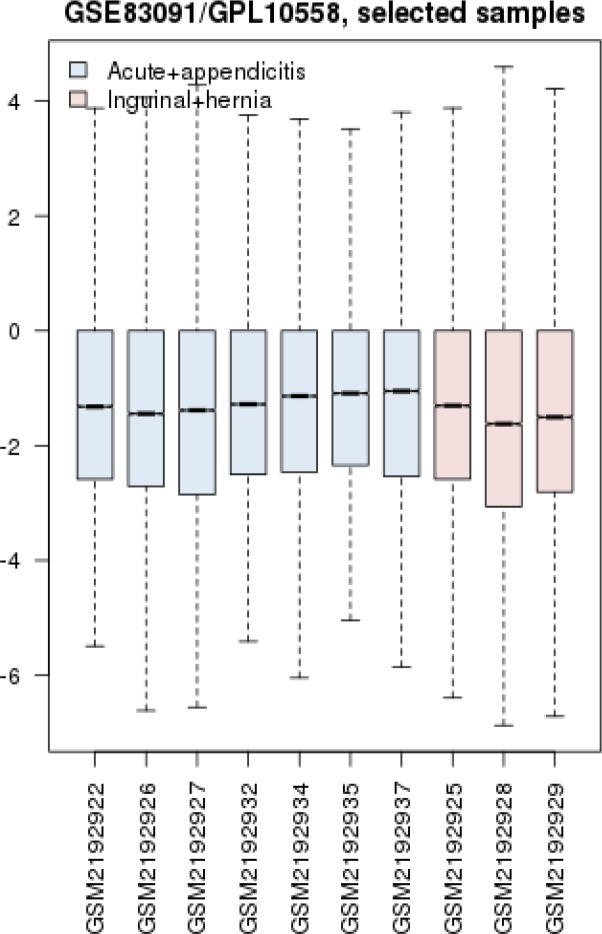
Box plot presentation of gene profiles of 3 acute appendicitis and 7 inguinal hernia samples. The analyzed profiles are comparable

**Figure 3:**
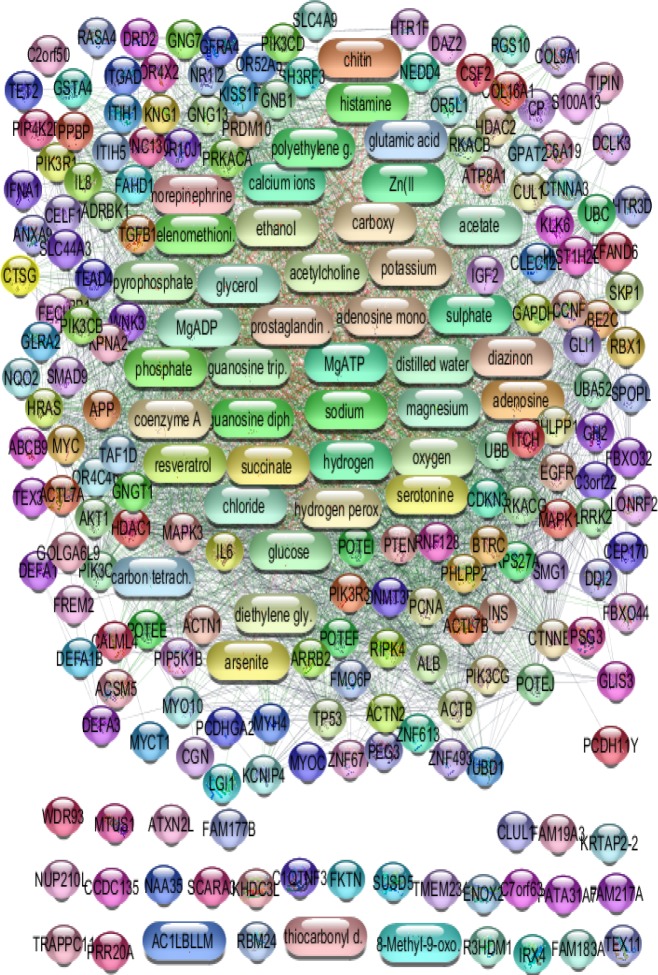
Network including Genes (round shape) and compounds (elongated shape) of appendicitis. The network contains 30 isolated nodes and a main connected component (196 nodes and 3317 edegs). Confidence score is 0.4

Gene expression profiles of 4 acute appendicitis patients and 17 inguinal hernia samples were compared via box plot analysis. The samples were not matched statistically (Figure 1) because profiles are not median centered. As it is shown in figure 2, the proper samples were selected for more analysis. Among top 250 significant DEGs, 146 Characterized ones with fold change; more than 2 and less than 0.5 were determined to include in network analysis. The 146 selected DEGs plus 100 relevant individual genes or compounds were organized by STITCH application of Cytoscape software as network of appendicitis. The network including 226 nodes was constructed (see figure 3). As it is appeared 20 queried DEGs were not recognized by STITCH. After network analysis, about 10% (20 nodes) of top nodes based on degree value were identified as hubs. As it is shown in the table 1, the introduced hubs are combination of proteins and compounds. All compounds content of network including 40 nodes are presented in the table 2. As it is appeared in the table 1, the top 9 hubs are compounds and are similar as top compounds in the table 2.

**Table 2:** All connected compounds in appendicitis network are presented. The colored ones are common with top nodes in table 1

R	Name	Degree	BC	CC
1	MgATP	111	0.044593	0.690141
2	glycerol	103	0.064153	0.671233
3	MgADP	103	0.033205	0.668942
4	calcium ions	97	0.039272	0.646865
5	chloride	91	0.040757	0.636364
6	magnesium	91	0.03123	0.636364
7	phosphate	86	0.014685	0.636364
8	sulphate	84	0.030589	0.622222
9	acetate	83	0.02295	0.622222
10	carboxy	77	0.011449	0.61442
11	sodium	77	0.031398	0.608696
12	distilled water	73	0.012154	0.603077
13	Zn(II	72	0.021814	0.593939
14	guanosine trip.	70	0.007893	0.590361
15	glucose	65	0.00962	0.573099
16	oxygen	65	0.007017	0.579882
17	polyethylene g.	64	0.008641	0.583333
18	hydrogen	62	0.01616	0.57478
19	hydrogen perox.	61	0.015314	0.578171
20	ethanol	58	0.007171	0.561605
21	potassium	58	0.007077	0.558405
22	adenosine	55	0.00411	0.561605
23	pyrophosphate	55	0.006656	0.556818
24	guanosine diph.	54	0.003892	0.545961
25	selenomethioni.	53	0.013893	0.54902
26	glutamic acid	52	0.002947	0.550562
27	histamine	49	0.003153	0.544444
28	prostaglandin .	48	0.001708	0.542936
229	succinate	48	0.002585	0.541436
30	adenosine mono.	46	0.004388	0.539945
31	acetylcholine	45	0.002846	0.531165
32	norepinephrine	43	9.56E-04	0.528302
33	chitin	41	0.003285	0.528302
34	coenzyme A	40	0.006368	0.521277
34	resveratrol	39	0.00398	0.514436
36	serotonine	38	0.001041	0.509091
37	arsenite	28	0.032664	0.509091
38	diethylene gly.	28	0.003629	0.483951
39	diazinon	17	0.011853	0.47343
40	carbon tetrach.	10	1.31E-06	0.446469

## Discussion

Network analysis of diseases is an attractable field which used to biomarker discovery and also gene screening of disorders (14-16). Suitable sample selection is an important step in research. As it was shown in the figures 1 and 2, boxplot analysis is a useful method to match samples (17). PPI network in figure 3 contains 226 proteins and compound that are involved in appendicitis. Main aim in this study is finding critical compounds that are involved in appendicitis. Network analysis led to introduce 20 hub nodes for appendicitis network (see table 1). Hub nodes include 6 protein and 14 compounds. All compounds of PPI network are 43 individual that three of them are isolated and the interacted ones (see table 1) are 40 individuals. Surprisingly 9 compounds including MgATP, glycerol, MgADP, calcium ions, chloride, magnesium, phosphate, sulphate, and acetate play role as top hub nodes, therefore they can be considered as key elements of network (18). As it is depicted in table 1, the key elements are top nodes based on betweenness centrality and closeness centrality values, however phosphate is an exception regarding betweenness centrality. Sodium the other hubs which is ranked in row 15 in table 1 and row 11 in table 2 can be ranked in row 6 in table 2 based on betweenness centrality. Since betweenness is an important centrality parameters (19), sodium can be considered as a key element. 

It can be concluded that there are 10 critical compounds which are involved in appendicitis. In following part we investigate possible relationships between the introduced 10 critical compounds and appendicitis in literature: Nusrat S. Shommu et al. evaluated blood samples of 32 non-perforated appendicitis children (NPAC) in comparison with 72 non- appendicitis children (NAC). The NAC were the patients with not appendicitis abdominal pain. This research group also examined urine of 66 NAC relative to 27 NPAC. 

As they are reported procalcitonin concentration in serum of NPAC group increases and acetate concentration level decreases (20). Calcitonin is hormone of thyroid gland and is responsible for decrement of calcium ion in blood. Mg up-regulates calcitonin production, therefore plays a central role in calcium ion in blood (21). Role of calcium in contraction of smooth muscles is known in details (22). Intra-abdominal muscle contraction is reported in appendicitis (23). As it is shown in the table 2, Calcium, magnesium, acetate are the three elements of central compounds related to appendicitis. MgATP and MgADP are the other two important compounds which are composites including Mg. Today it is well-known that calcium regulation is correlated to phosphate homeostasis (24). An investigation showed that there is correlation between appendicitis and oxidative stresses. Since glycerol increases oxidation reaction (25) it can be related indirectly to appendicitis (26). 

It seems that appendicitis is correlated mainly to ion compounds especially calcium. It is a logical expectation that calcium level of blood be altered significantly. The finding s are in agreement with this point that calcium and the other agents that are involved in calcium concentration regulation are the important compounds which are possible biomarkers for acute appendicitis. As it is appeared in the table 2, concentration alteration of other metal ions such as sodium, zinc, and potassium are related to appendicitis. Since flame photometry is a simple method for detection metal concentration it can be concluded that differentiation between appendicitis pain and the other abdominal pains is possible. However, more investigations are needed to validate this finding.

Based on our findings a ten member panel of compounds including calcium play critical role in differentiation of appendicitis of the other non-appendicitis abdominal pains. Therefore, it is possible to introduce a simple laboratory method for diagnosis appendicitis. In this regard more investigations to validate the findings are required.
